# Effect of Exercise on Arterial Stiffness in Healthy Young, Middle-Aged and Older Women: A Systematic Review

**DOI:** 10.3390/nu15020308

**Published:** 2023-01-07

**Authors:** Yong Sheng Lan, Teng Keen Khong, Ashril Yusof

**Affiliations:** 1 Faculty of Sports and Exercise Science, Universiti Malaya, Kuala Lumpur 50603, Malaysia; 2 School of Physical Education, Changchun Normal University, Changchun 130032, China

**Keywords:** exercise, arteries, women

## Abstract

Arterial stiffness, an age-dependent phenomenon, is improved with exercise, which in turn may prevent cardiovascular diseases in women. However, there is a lack of consolidated information on the impact of exercise on arterial stiffness among healthy women. The aim of this review was to (i) analyse the effect of exercise on arterial stiffness in healthy young, middle-aged, and older women, and (ii) recommend types, intensity, and frequency for each age group. Database searches on PubMed, ScienceDirect, Web of Science, and Scopus were conducted using PRISMA guidelines until September 2022. The keywords were: exercise, women/female, and arterial stiffness. The inclusion criteria were: healthy women, supervised exercise, and arterial stiffness measures. Study quality and bias were assessed using the PEDro scale. Fifty-one papers were classified into young (n = 15), middle-aged (n = 14), and older (n = 22) women. Improvements in arterial stiffness were observed among: young women (Pulse Wave Velocity, PWV: 4.9–6.6 m/s), following an 8-week high-intensity aerobic (3 days/week) or hypoxic high-intensity interval training; middle-aged women (PWV: 5.1–7.9 m/s), aerobic exercise with moderate intensity or stretching exercise at “moderate to heavy” (Borg Scale), 20–30 s per site, 10 s of rest interval for 30 min; and for older women (PWV: 7.9–15.6 m/s), resistance training at light intensity, aerobic exercise at any intensity, or a combination of the two exercises. This review shows that arterial stiffness increases with age in healthy women and has an inverse relationship with exercise intensity. Therefore, when prescribing exercise to improve arterial stiffness, age and arterial stiffness measures should be accounted for.

## 1. Introduction

Arterial stiffness describes rigidity in the arterial wall, which has been accepted as an independent risk factor for cardiovascular morbidity and mortality [1]. Increased arterial stiffness is also highly associated with an increased risk of cardiovascular diseases (CVDs), such as hypertension [2], chronic kidney disease [3], and stroke [4]. The risk of CVDs in women is often underestimated due to the misconception that women are more “protected” than men [5] and is, hence, understudied, under-recognised, underdiagnosed, undertreated, and under-represented [6]. The Lancet reported that 35% of deaths in women worldwide are caused by CVDs [6], with a staggering 275 million women diagnosed, while 8.9 million died from CVDs in 2019. The prevalence of high arterial stiffness was 10.6% for ages under 29 years, 40.1% for ages 30–49 years, and 91.5% for ages over 50 years [7]. Thus, to delay the development of atherosclerotic CVDs, prevention or containment of risk factors that accelerate arterial stiffness should be in place [8], considering that the etiological factors contributing to arterial stiffness among women in different age groups may differ [9]. Exercise is the most common modality in treating and preventing arterial stiffness [10]. Therefore, exercise prescriptions should consider the etiological factors of women at different ages to improve arterial stiffness more effectively.

Arterial stiffness is an unavoidable physiological process. It is difficult to determine which factors contribute to the progression of arterial stiffness. Therefore, the current interventions for arterial stiffness suffer from a lack of targeting. This may be the reason why medical treatment for the progression of arterial stiffness is modestly effective [3]. In fact, specific populations, such as young women, middle-aged women, and older women, have specific factors associated with arterial stiffness. Oestrogen is thought to play an important role in the female-specific changes in arterial stiffness [9]. In adult women, overall oestrogen levels reach a peak in the late 20s. Then oestrogen levels begin to decline at 30 years of age until menopause. Menopause is a milestone in female ageing- it means that oestrogen levels will remain at low levels [11]. Under this premise, young women are in a period of oestrogen upswing, middle-aged women are mainly in a period of oestrogen downturn, and older women are in a period of low oestrogen levels. Oestrogen could reduce arterial stiffness through increased nitric oxide (NO) [12]. In addition, norepinephrine, which can drive vascular smooth cell (VSMC) contraction, is positively correlated with age in women [13]. VSMCs are most abundant cell types in vessel walls and have emerged as key players in arterial stiffness [14]. Furthermore, the advanced glycation end product (AGE) accumulation hastens multisystem functional decline with age [15]. AGEs are a non-enzymatic process involving glycation of sugars or reactive oxoaldehyde with proteins, lipids, or nucleic acids. AGEs promote arterial stiffness through the upregulation of inflammation and collagen cross-linking [16], which, in turn, leads to endothelial dysfunction and subsequently increases vascular stiffness. Exercise can modulate these etiological factors and, thus, improve arterial stiffness. However, due to the different characteristics of these etiological factors in women of different ages, it is possible that similar exercise prescriptions may have different effects on arterial stiffness. Incidentally, there are studies that have tentatively confirmed this speculation. For example, an incremental exercise program to exhaustion using Bruce protocol does not modify arterial stiffness in young women [17] but increases arterial stiffness in middle-aged women [18]. In addition, resistance training at 80% 1-RM showed positive effects on arterial stiffness in young women [19] but no change in arterial stiffness in middle-aged and older women [20,21]. Considering the etiological factors and impact of exercise (types, intensity, and frequency), however, to date, no review has been conducted on the effects of exercise on arterial stiffness in healthy young, middle-aged, and older women.

The purpose of this review was to explore the current literature on the effects of exercise on arterial stiffness in healthy young, middle-aged, and older women, to provide guidance on exercise for the prevention of cardiovascular disease in women and propose suggestions for further research.

## 2. Methods

### 2.1. Data Selection

This systemic review was conducted in accordance with the Preferred Reporting Items for Systemic Reviews and Meta-analyses (PRISMA) Guideline [22]. The journal articles were searched electronically using the following databases: PubMed, ScienceDirect, Web of Science, and Scopus. Peer-reviewed articles published in English until 30th September 2022 were reviewed. No contact with the studies’ authors was made. The search strategy used in Web of Science is displayed in Table 1, and a similar strategy was used to search the other databases.

### 2.2. Studies Selection

**Figure 1:**
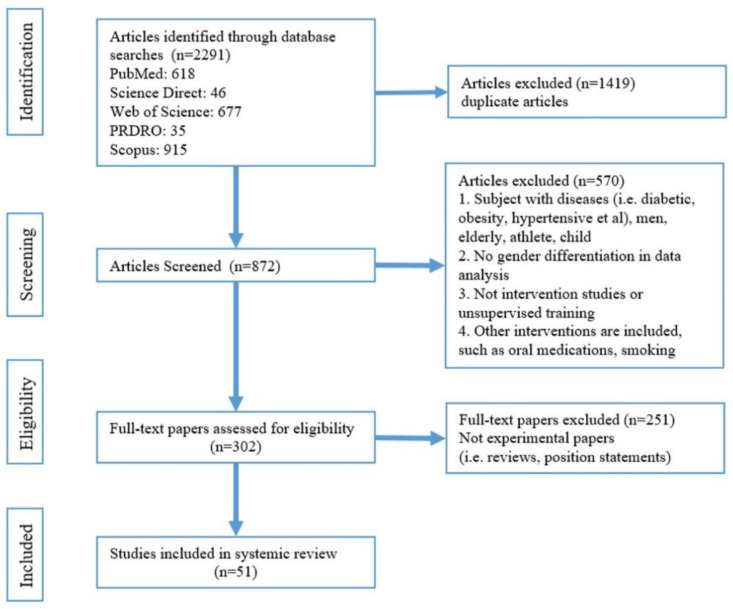
Flowchart for study selection.

Figure 1 shows a flow diagram of the search. The articles were selected for review after thorough screening and exclusion of ineligible articles. The inclusion criteria were as follows: exercise was the only intervention and must be under supervision; arterial stiffness assessment must be included; subjects were healthy women, without hypertension, diabetes, obesity, or pregnancy; statistics must be gender-specific and a research paper. Then, all the selected papers were categorised into a young-women group (Table 2), a middle-aged-women group (Table 3), and older-women group (Table 4). Women between 15 and 24 years of age are defined as young age [23], those aged more than 60 years or postmenopausal are defined as older women [24]. Women between the ages of young and older are middle-aged women.

### 2.3. Data Extraction and Studies Methodological Quality

The titles and/or abstracts of all articles in the database were reviewed to assess the requirement for full text based on the study selection criteria. Each full-text paper was evaluated systematically according to: (a) subject characteristics, (b) exercise volume (frequency, intensity, time, mode, etc.), and (c) main findings. Characteristics of the included studies for young, middle-aged, and old women are presented in Table 2, Table 3 and Table 4, respectively.

The quality assessment was performed using the PEDro Scale. Two reviewers independently assessed the articles and average score from both assessments was used. The 51 studies averaged a score of 7.96, with four studies scoring less than 7, mainly due to the absence of a double-blind design.

## 3. Results and Discussions

This systematic review aims at elucidating the effect of exercise on arterial stiffness measures (PWV, elastance) among young (18–24 years), middle-aged (25–49 years), and older (50 and above) women. Evidence from the retrieved studies (n = 51) suggests that exercise can be an effective way to improve arterial stiffness in women. In young and middle-aged women, high-intensity aerobic exercise and stretching at “somewhat heavy” to “heavy” based on the Borg Scale are recommended, while in older women, aerobic exercise at any intensity, resistance exercise at low intensity, combined aerobic and resistance exercise, and stretching and vibration training can all reduce arterial stiffness. In addition, this review also confirms that arterial stiffness is age-dependent in women. The pulse wave velocity (PWV), a wide measure of arterial stiffness, at rest ranges from 4.9–6.6 m/s and 5.1–7.9 m/s to 7.9–15.6 m/s in young, middle-aged, and elderly women, respectively. The values seem to increase slightly in middle-aged women whilst showing a sharp increase in older women compared to younger. These may influence the effect of exercise on arterial stiffness, where improvements in PWV are 7 and 8.7% in young and middle-aged women, respectively [35,42], while 12.5% in older women [51]. It appears that the effect of exercise is dependent on the severity of the arterial stiffness, which is, in turn, age-dependent. Factors, such as exercise prescription, i.e., type, intensity, duration, and frequency, are also major determinants for the said effects.

### 3.1. Defining Exercise Intensity

Exercise intensity is the primary factor in exercise prescription. Exercise intensities in this review are classified using HRmax, HRR, RM, METS, and VO_2_max. For HRmax: 50–63% is classified as light intensity; 64–76% as moderate intensity; and 77–93% as high intensity [70]. For HRR, light intensity and moderate intensity are defined as 30 and 50% HRR, respectively, while high intensity is defined as more than 50% HRR [71]. For RM, less than 67% of 1 RM or more than 12 RM is classified as low intensity [72], 67–85% of 1 RM or 6–12 RM as moderate intensity, while more than 85% of 1 RM or less than 6 RM is classified as high intensity. For METS, light intensity is less than 3 METs; moderate intensity ranges from 3 to 6 METs; and high intensity is more than 6 METs [73]. For VO_2_max, less than 51% is defined as light intensity; between 52 and 67% is defined as moderate intensity; higher than 67% is defined as high intensity [74].

### 3.2. Effect of Exercise on Arterial Stiffness in Young Women

Among young women, five different types of exercises, aerobic, HIIT, resistance training (RT), anaerobic, and stretching exercises have been reported in 15 studies. However, only two studies have shown the positive effects of aerobic (running, cycling, or elliptical) [35] and hypoxic HIIT-running [25] exercises on arterial stiffness (about 7% improvements). In these studies, moderate–high intensity was based on HRmax and HRR respectively. The lack of changes in other studies [17,29,34], which also employed aerobic exercises, could be due to (i) vascular physiology, (ii) the acute nature of exercise intervention compared to 3 days/week for 8 weeks in Lane et al. [35] and 6 weeks in Park et al. [25], and (iii) environment (hypoxic) [25]. On the other hand, while RT is the most widely studied type of exercise [21,28,30,31,32,33,37], no changes in arterial stiffness were reported. In these studies, moderate–high intensity based on RM was employed. Adversely, acute-effect moderate–high intensity RT has shown a transient increase in PWV [28,31,32,33]. The increase in arterial stiffness due to resistance training may be due to increased levels of catecholamines and activation of the sympathetic nervous system [75]. Catecholamine levels and the sympathetic nervous system can increase vascular smooth muscle tone and, thus, vascular stiffness through humoral and neural regulation, respectively [75].

Based on the evidence presented, to observed changes in arterial stiffness among young women, at least 3 days/week of 8-week high-intensity aerobic (running or cycling) or hypoxic HIIT training are recommended, while acute aerobic or resistance-type exercise is not recommended.

### 3.3. Effect of Exercise on Arterial Stiffness in Middle-Aged Women

Among middle-aged women, four different types of exercises, including aerobic, RT, stretching exercise, and myofascial release exercises, are reported in 14 studies. For aerobic exercise, out of four, three studies [18,40,46,47] showed positive effects on arterial stiffness (about 23.27% improvement). It appears that acute high-intensity running exercise based on HR and HRR [18,40] and prolonged moderate cycling exercise based on VO_2_max with 2 days/week for 12 weeks [46] are beneficial for middle-aged women. One study [47] did not observe any change following a 14-week high-intensity cycling exercise based on HRR, which could be due to (i) study design (cross-over with insufficient wash-out period and small sample size), (ii) excess intensity, and (iii) low mean age. The three studies which showed a positive effect from aerobic exercise on arterial stiffness in middle-aged women are at moderate–high intensities. In addition, no adverse effect of aerobic exercise on arterial stiffness was reported in middle-aged women.

Meanwhile, for RT, which is the most commonly studied type of exercise among middle-aged women, there are two studies [41,45] that reported positive effects on arterial stiffness in middle-aged women (about 10.97% improvement), while five others do not [20,38,39,46,48]. The lack of changes following RT in this age group could be due to multifactorial reasons, (i) exercise intensity, (ii) mode of contraction, and (iii) muscle group tested. It seems that acute low–moderate RM (Augustine et al., 2018) and prolonged training (2 days/week for 10-week; in Okamoto et al. 2009), involving major muscle groups (upper limb) and using isotonic contraction, are important factors to consider in prescribing exercise to middle-aged women.

Although low–moderate intensity RT based on RM and body weight showed a positive effect on arterial stiffness [41,45], several other studies reported that light-intensity RT based on RM has either a detrimental effect or no effect on vascular health [38,46,48], while high-intensity RT with maximal isokinetic knee extension/flexion also has a negative effect on arterial stiffness [39] in middle-aged women. In addition, to a lesser extent, the menstrual cycle could also influence the effect of exercise on arterial stiffness among middle-aged women [41].

While for stretching exercise, two studies [42,43] showed positive results (about 9.2% improvement), one study does not report positive changes [44]. The lack of change could be associated with low stretching activity, stretching duration per site, and interval of stretching may be a key factor influencing the change in arterial stiffness. Based on the positive findings [42,43], stretching intensity of “somewhat heavy” to “heavy” in Borg Scale 7 days/week for 6 months [43], stretching exercise with 20–30 s per site, and 10 s of rest interval for 30 min [42] may be helpful for vascular health among middle-aged women.

In brief, the most preferable type of exercise is aerobic exercise at moderate–high-intensity running or cycling, acute or prolonged (2 days/week for 12 weeks), while for RT, low–moderate-intensity acute or prolonged (2 days/week for 10 weeks) involving major muscle groups and using the isotonic mode of contraction, and for stretching exercise, acute 30 min or prolonged at “somewhat heavy” to “heavy” based on Borg Scale seem beneficial to improve arterial stiffness among middle-aged women.

### 3.4. Effect of Exercise on Arterial Stiffness in Older Women

Among older women, there are a total of 22 studies on the effect of exercise on arterial stiffness, the highest number compared to young women and middle-aged women. The most common types of exercise reported are aerobic exercises (walking, running, cycling, aquarobics, Taichi, steps), prolonged aerobic [51,52,53,56,57,58,60,62,63,64,66,68,69] and acute aerobic [56,64], resistance training [21,50,54,55,61,68], combined resistance training and aerobic exercise [49,59,65,67], flexibility training [61], and vibration training [50]. The majority of the studies (17) showed a positive effect of exercise on arterial stiffness in older women. More importantly, no adverse effect of exercise was reported in the studies. Hence, the types of exercises listed (excluding vibration training) can be recommended for older women depending on their ability.

For aerobic exercise, there are 16 studies listed, with all reporting positive changes in arterial stiffness among older women (about 18.46% improvement). Out of these, 14 studies prescribed prolonged exercises, while 2 prescribed acute types. The intensity of the aerobic exercise ranges from low to moderate to high based on HRmax (60–80%), HRR (40–80%), METS (1.5–3.0), VO_2_peak (50%), and at lactate threshold, while the duration of the intervention ranges from 8 to 18 weeks with 1–3 days/week in most studies. Meanwhile, two studies reported positive changes following acute aerobic exercise. The intensity of these exercises ranges from moderate to high based on self-paced walk (1 h walk) [56] and anaerobic threshold cycling (90%) [64]. Based on the evidence presented, it is suggested that for a long-term effect, any intensity, 8–18 weeks, 1–3 days/week, while for the acute effect of aerobic exercise, 20–90 min moderate to high intensity can be considered as the ideal type of aerobic exercise (walking, running, cycling) to be prescribed for older women.

For RT, vascular stiffness improvements in older women were observed following low-intensity exercises based on RM (about 0.85% improvement) [50,61]. What’s more, no studies have found a detrimental effect of low-intensity resistance training on the vascular health of older women as typically seen in middle-aged women. Therefore, older women can consider low-resistance training based on their ability. In addition, some researchers found that combining low-resistance training and aerobic exercise also showed a beneficial effect on vascular stiffness in older women [49,50].

For stretching exercise, 2 days/week for 16 weeks performing a whole-body static stretching program may be helpful for vascular health among middle-aged women. For vibration exercise, dynamic leg exercises (full squats, high squats, wide squats, and calf raises) on a vibrating platform with 24–40 Hertz intensity for 12 weeks showed no change in arterial stiffness. Therefore, it is difficult to provide this exercise program for this age group.

In conclusion, the most preferable type of exercise is aerobic exercise, such as prolonged swimming, rotating exercise, chair-based exercise, walking, cycling, Taichi, steps with low–moderate–high intensity (1–3 days/week, 8–18 weeks), or acute walking/cycling with moderate–high intensity (20–90 min). For RT, low intensity or low-intensity RT combined with aerobic exercise, and for stretching exercise, 2 days/week for 16 weeks involving a whole-body static stretching program seem beneficial to improve arterial stiffness among older women.

### 3.5. Possible Mechanisms Underlying the Effect of Exercise on Arterial Stiffness in Women

Acute and long-term exercises have shown beneficial effects on arterial stiffness in women in general. However, the changes following acute exercise may only be temporary, as arterial stiffness decreased significantly after acute exercise and would return to basal levels 24 h later [76,77,78]. These temporary changes may be exclusive to improved vascular function rather than structural adaptations [79,80]. Acute exercise increases blood flow and shear stress, which leads to an increased release of endothelial NO, further causing the vascular smooth muscle to relax in response to the sustained stress caused by increased blood flow [81] and sympathetic excitability [82], which may account for the decrease in arterial stiffness. There are also other mechanisms by which acute exercise alters vascular stiffness, such as reduced release of vasodilator mediators, vasoconstrictor factors, or vascular modifications [83].

On the other hand, long-term exercise causes and an adaptive response in the arteries. This could be due to the adaptation of the improved endothelial function [84], upregulation of endothelial NO synthase protein expression and phosphorylation, reduction in matrix metalloproteinases [85], improvement in insulin resistance [86], a decrease in proinflammatory cytokines [87], and improvement in the ability of glucose tolerance and insulin sensitivity [87]. Another important biomarker is the glycation index, which increases with age. The products of glycation, AGEs, have negative effects on vascular media and adventitia layers, which will cause the vasculature to be stiffer. High AGE levels may be a biomarker contributing to reduced physical activity in an older population [88]. Hence, regular exercise may improve arterial stiffness by reducing the production of glycation in females [89].

### 3.6. General Guidelines and Direction for Further Studies Investigating the Effect of Exercise on Arterial Stiffness in Women

It is clear that, while the effects of exercise on arterial stiffness in older women have been studied in more detail, relatively less research has been carried out on its effects in young and middle-aged women. Therefore, fewer exercise prescriptions could be presented to young and middle-aged women compared with older women. However, it is important to know that arterial stiffness in older women is unlikely to be restored to the level of young women, even when significant improvements have been made through exercise. Therefore, it should be highlighted that the greatest chance of eradicating cardiovascular disease in the future may be in primary and elementary prevention that begins at a young age [90].

Based on this review, it seems that arterial stiffness is more challenging to regulate through exercise in young and middle-aged women compared to older women. This may be attributed to the fact that older women tend to have stiffer arteries and a greater range of adjustment. Each of the principles of exercise prescription, including intensity, frequency, and time, can individually influence the effect of exercise on arterial stiffness (see *What are the new findings* for some practical applications). Future research should conduct dose-dependent intervention studies between each principle of exercise prescription and arterial stiffness in young and middle-aged women, especially focusing on exercise intensity due to its greater criticality than other principles. Indicators to assess intensity should be matched to the type of exercise, such as HRmax, HRR, and VO_2_max can assess aerobic exercise and HIIT intensity, while RM can assess resistance training intensity. In addition, both acute and regular exercise interventions should be performed to reveal the effect of exercise more precisely on arterial stiffness. Acute exercise may only cause functional changes in the arteries, whereas regular exercise may trigger alterations in the structure of the arteries. For acute exercise, arterial stiffness measurements should be taken at multiple time points, including before exercise, 15 min after exercise, and 30 min after exercise, to reflect the dynamic changes in arterial stiffness. Similarly, for regular exercise, multiple time-point measurements of arterial stiffness are also necessary. However, a sufficient period of intervention is more crucial and, according to this review, at least 8 weeks of exercise intervention are required. For older women, there are more exercise prescriptions available to improve arterial stiffness than for young and middle-aged women. Therefore, future research could focus more on developing types of exercise that are preferred by older women. In addition, arterial stiffness may be influenced by some confounding factors, such as somatotype, lifestyle, and diet. Stiffer arteries are found in physically inactive and obese people [91], while high sodium intake contributes to arterial stiffening [92]. Further research could take these factors into account to provide more effective guidelines for improving arterial stiffness in women.

## 4. Summary

### 4.1. What Is Already Known?

- Arterial stiffness is an independent predictor of cardiovascular disease.
- Arterial stiffness increases with age in women.
- Exercise can improve arterial stiffness in pathological states.

### 4.2. What Are the New Findings?

- The effect of exercise is dependent on age and arterial stiffness measure.
- Exercise intensity is inversely related to age and arterial stiffness measure.
- For young women, prolonged high-intensity aerobic exercise is recommended.
- For middle-aged women, moderate-intensity aerobic or stretching exercises are recommended.
- For older women, any intensity of aerobic exercise, such as daily walking and cycling, or light-intensity resistance training are recommended.
- To summarise, structured exercises can influence arterial stiffness positively. In addition, aerobic exercise is consistently found to be beneficial for woman, with an inverse relationship between age and recommended aerobic exercise intensity.

## Figures and Tables

**Table 1:** Search strategy for Web of Science.

Filter:	(English) AND (in Title and Abstract)	Results
#1	(exercise) and (women) and (arterial stiffness)	549
#2	(exercise) and (female) and (arterial stiffness)	182
#3	Total after merging duplicate articles from #1 and #2	677

**Table 2:** Characteristics of selected experimental trials on young women (n = 15).

Reference	Subjects’ Characteristics	Exercise Prescription			Main Findings
		Type	Duration/Frequency	Intensity	
Park et al. (2022) [25]	Normoxic group; N = 10; Body Mass Index (BMI) = 19.1 ± 1.2 mkg^−2^ Hypoxic group; N = 10; BMI = 19.0 ± 1.1 mkg^−2^ All aged = 24.85 ± 3.84 yo	High-intensity interval Training (HIIT)	90-min, 3 day/week for 6 weeks	90–95% Heart rate (HR)max	Normoxic group showed no change in PWV. Hypoxic group showed reduction in PWV
Yan et al. (2021) [26]	African Americans (AA); N = 8; aged = 22 ± 1.0 yo; BMI = 24.7 ± 0.8 Caucasian Americans (CA); N = 12; aged = 22 ± 1.0 yo; BMI = 22.9 ± 0.7	Anaerobic exercise	30-min maximal anaerobic exercise (Exercise 1) with 30 min of recovery, following a maximal anaerobic exercise (exercise 2)	Maximal intensity	No changes in PWV. AA: Rest: 5.2 ± 0.2 m/s; 5 min post exercise 1: 5.0 ± 0.2 m/s; 15 min post exercise 1: 5.2 ± 0.3 m/s; 30 min post exercise 1: 5.1 ± 0.2 m/s; 5 min post exercise 2: 5.0 ± 0.3 m/s; 15 min post exercise 2: 5.2 ± 0.4 m/s; 30 min post exercise 2: 5.1 ± 0.3 m/s; CA: Rest: 4.9 ± 0.2 m/s; 5 min post exercise 1: 4.9 ± 0.2 m/s; 15 min post exercise 1: 5.2 ± 0.4 m/s; 30 min post exercise 1: 4.9 ± 0.4 m/s; 5 min post exercise 2: 4.9 ± 0.2 m/s; 15 min post exercise 2: 5.1 ± 0.3 m/s; 30 min post exercise 2: 4.9 ± 0.2 m/s
Lee & Lee (2021) [27]	N = 10; aged = 23.20 ± 0.59 yo; BMI = 25.2 ± 1.3 mkg^−2^	Foam flexibility training	30-min	Bodyweight to apply pressure	No changes in PWV.
Marshall et al. (2021) [28]	N = 10; aged = 22 ± 2.0 yo; BMI = 22.8 ± 2.2 mkg^−2^	Resistance training (HI-BRE)	6 set × 15 s	HR 180 bpm	Increase in PWV Rest: 5.4 ± 0.8 m/s; 10 min post exercise: 5.7 ± 1.0 m/s; 30 min post exercise: 5.4 ± 0.7 m/s; 60 min post exercise: 5.1 ± 0.5 m/s
Lim et al. (2018) [29]	N = 15; aged = 21.4 ± 0.7 yo; BMI = NA	HIIT vs. Aerobic exercise	HIIT: 40-min Aerobic: 47-min	HIIT at 90% HRmax Aerobic at 70% and 50% HRmax	HIIT group no change in PWV Aerobic: No change in PWV Rest: 5.5 ± 0.1 m/s; post-exercise: 5.5 ± 0.1 m/s
Tomschi et al. (2018) [30]	Upper body training group N = 10; aged = 21.5 ± 1.4 yo; BMI = 22.1 ± 2.0 mkg^−2^ Lower body training group N = 10; aged = 22.3 ± 1.30 yo; BMI = 23.3 ± 2.4 mkg^−2^	Resistance exercise	Resistance exercise 4 movement × 3 set × 12 rep	At 70% of 1 RM	No change in PWV in both groups
Augustine et al. (2018) [31]	N = 13; aged = 24 ± 4.0; BMI = 22.0 ± 3.1 mkg^−2^	Resistance training	5 set × 5 rep bench press and 5 set × 10 rep bicep curl	5 RM for bench press and 10 RM for bicep curl	Increase in PWV Rest: 5.1 ± 0.5 m/s; 10 min post exercise: 6.1 ± 0.8 m/s; 20 min post exercise: 5.7 ± 0.4 m/s
Okamoto et al. (2017) [32]	N = 9; aged = 21.30 ± 0.8 yo; BMI = NA	Resistance training	5 set × 5 rep bench press and 5 set × 10 rep bicep curl	80% 1 RM for bench press followed 70% 1 RM for bicep curl	Follicular phase showed an increase in PWV: increased from baseline by 10 ± 10% (Δ94 ± 91 cm/s) and 8 ± 9% (Δ74 ± 78 cm/s) at 30 and 60 min post exercise Luteal phase showed no change in PWV
Kingsley et al. (2017) [33]	N = 12: aged = 23 ± 4.0 yo; BMI = 24.3 ± 4.7 mkg^−2^	Resistance training	3 set × 10 rep of bench press and 3 set × 10 rep of dead lift	At 75% of 1 RM	Increase in PWV Rest: 5.1 ± 0.8 m/s; post exercise: 5.6 ± 0.7 m/s
Perdomo et al. (2016) [34]	N = 15; aged = 24.3 ± 3.0 yo; BMI = 23.4 ± 2.6 mkg^−2^	Aerobic exercise	30 min run	At 70–75% of HRmax	No difference in PWV Rest: 5.69 ± 0.64 m/s; post exercise: 5.62 ± 0.7 m/s
Lane et al. (2014) [35]	N = 25; aged = 24 ± 1.0 yo; BMI = 25.0 ± 4.0 mkg^−2^	Aerobic exercise	30–60 min for 3 days/week, 8 weeks	At 60–90% of HRmax	Decrease in arterial elastance Rest: 5.7 ± 1.2 m/s; post exercise: 5.3 ± 0.7 m/s
Harris et al. (2014) [36]	Interval training group; N = 6; BMI = 23.6 ± 1.8 mkg^−2^ Continuous training group; N = 6; BMI = 23.1 ± 2.6 mkg^−2^ All aged = 22 ± 2.0 yo;	Anaerobic sprint cycling	3 days/week for 4 weeks	Maximum	No change in PWV Interval training group: Rest: 6.0 ± 0.8 m/s; post exercise: 6.2 ± 0.5 m/s Continuous training group: Rest: 6.6 ± 0.8 m/s; post exercise: 7.4 ± 0.7 m/s
Rossow et al. (2014) [21]	N = 16; aged = 22 ± 2.0 yo; BMI = NA	Resistance training	6 movement × 3 set × 10 for 3 days/week, for 8 weeks	At 80% of 1 RM	No change in PWV
Doonan et al. (2013) [17]	N = 55; aged = 23.7 ± 4.80 yo; BMI = 21.7 ± 2.1 mkg^−2^	Aerobic exercise	To exhaustion	Maximum, graded intensity (Bruce protocol)	No change in PWV
Okamoto et al. (2006) [37]	Sedentary group; N = 9; aged; 19.9 ± 1.20; BMI = 20.4 ± 3.1 mkg^−2^ Eccentric training group; N = 10; aged = 18.9 ± 0.30 yo; BMI = 21.7 ± 2.1 mkg^−2^ Concentric training group, N = 10; aged = 19.1 ± 0.30 yo; BMI = 21.9 ± 3.1 mkg^−2^	Resistance training	5 set × 10 rep of arm curl for 3 days/week 8 weeks	Eccentric group at 100% of 1 RM, and Concentric group at 80% of 1 RM	Eccentric group remain unchanged in PWV but increased after detraining. Concentric group increased in PWV and returned to bassline after detraining.

BMI: Body mass index; HR: Heart-rate; HIIT: High-intensity interval training; RM: One-repetition maximum; HRmax: Maximal heart rate; HRR: Heart rate reserve; HI-BRE: High-intensity battling rope exercise; PWV: Pulse wave velocity; CAVI: Cardio-ankle vascular index.

**Table 3:** Characteristics of selected experimental trials in middle-aged women (n = 14).

Reference	Subjects’ Characteristics	Exercise Prescription			Main Findings
		Type	Time/Frequency	Intensity	
Cebrowska et al. (2022) [38]	N = 22; aged = 35.4 ± 12.3 BMI = 26.0 ± 4.2 mkg^−2^	Resistance training (IHG)	3-min	30% maximal IHG strength	Increase in ASI
Lee & Lee (2021) [27]	N = 10; aged = 44.50 ± 0.91 yo; BMI = 23.5 ± 0.8 mkg^−2^	Flexibility training	30-min	Bodyweight to apply pressure	No changes in PWV
Grigoriadis et al. (2020) [39]	N = 17,; aged = 25 ± 4 yo; BMI = 23.5 ± 4.2 mkg^−2^	Resistance training	3 set × 10 rep	Maximal isokinetic knee extension/flexion	Increase in PWV Rest: 5.1 ± 0.4 m/s; 5 min post exercise: 5.2 ± 0.4 m/s; 30 min post exercise: 5.2 ± 0.3 m/s
Sun et al. (2020) [40]	N = 29; aged = 27 ± 5 yo; BMI = 22.1 ± 2.8 mkg^−2^	Aerobic exercise	45-min	70% HRR	Decrease in PWV;
Augustine et al. (2018) [41]	N = 18; aged = 28 ± 7 yo; BMI = 22.6 ± 2.9 mkg^−2^	Resistance training	5 set of 5 RM on the bench press then 5 set × 10 rep of biceps curl at different menstrual cycle phases	5 RM and 10 RM	Increase in central PWV during early luteal phase (LP). Increase in peripheral PWV during follicular phase (FP). Decrease in central PWV during FP. Rest: 7.9 m/s; post exercise: 6.7 m/s Decrease in peripheral PWV during early LP. Rest: 7.9 m/s; post exercise: 6.7 m/s
Logan et al. (2018) [42]	N = 30; aged = 44.37 ± 10.8 yo; BMI = NA	Static Stretching	Repeated 3 to 5 times with 10 s of rest 30 min	“somewhat heavy” to “heavy” in Borg Scale	Decrease in PWV; Rest: 6.93 ± 1.54 m/s; post exercise: 6.29 ± 1.17 m/s
Shinno et al. (2017) [43]	N = 21; aged = 47.9 ± 2.2 yo; BMI = 21.6 ± 4.3 mkg^−2^	Static Stretching	20–30 s per site 7 days/week 6 months	Whole-body static stretching	Decrease in PWV;
Li et al. (2015) [18]	N = 18; aged = 25.5 ± 2.8; BMI = NA	Aerobic exercise	To exhaustion	Maximum, graded intensity (Bruce protocol)	Decrease in arterial elastance Rest: 8.68 ± 1.85 m/s; During exercise: 13.25 ± 3.92 m/s; post exercise: 10.70 ± 4.40 m/s
Kim et al. (2012) [44]	Exercise group; N = 16; aged = 45.7 ± 1.0 yo; BMI = 26.0 ± 1.0 mkg^−2^ Control group; N = 18; aged = 43.2 ± 1.0 yo BMI = 27.0 ± 1.0 mkg^−2^	Stretching (Hata Yoga exercise)	60-min; 2 days/week 8 months	60–80% MHR	No change in arterial compliance
Fjeldstad et al. (2009) [20]	Training group; N = 21; aged = 33.2 ± 2.1 yo; BMI = 26.6 ± 1.4 mkg^−2^ Control group; N = 11; aged = 36.8 ± 3.2 yo; BMI = 24.4 ± 3.1 mkg^−2^	Resistance training	30 min of 7 movements × 2–3 sets (8 reps) 12 weeks	At 80% of 1 RM	No change in arterial compliance Rest: 7.6 ± 0.5 mL/mmHg × 100; post exercise: 7.8 ± 0.6 mL/mmHg × 100
Okamoto et al. (2009) [45]	N = 12; aged = 42–55 yo; BMI = 23.6 ± 1.0 mkg^−2^	Resistance training	40-min, 6 movements × 2 sets (12–15 reps) × 2 days/week for 10 weeks. Between each set 10 min walk.	Body weight and light dumbbells (500–1000 g).	Decrease in PWV Rest: 1270 cm/s; post exercise: 1175 cm/s
Yoshizawa et al. (2009) [46]	N = 35, Resistance training group; Aged = 47 ± 2 yo; BMI = 21.6 ± 4.3 mkg^−2^; Aerobic exercise group; Aged = 47 ± 2 yo; BMI = 24.6 ± 1.1 mkg^−2^; Control group; Aged = 49 ± 3; BMI = 21.8 ± 1.0 mkg^−2^;	Resistance training Aerobic exercise	6 movements × 3 sets (10 reps) × 2 days/week for 12 weeks. 30 min × 2 days/week cycling for 12 weeks.	Resistance: 60% 1 RM Aerobic: 60–70% VO_2_max	No change in PWV for Resistance group. Decrease in PWV for Aerobic group.
Sabatier et al. (2008) [47]	N = 13; aged = 33 ± 4 yo; BMI = 29.1 ± 9.1 mkg^−2^; High intensity group vs. Low intensity (cross-over)	Aerobic exercise	50 min cycling × 2 days/week 14 weeks	High intensity: 75–90% HRR Low intensity: 55–65% HRR	No change in PWV for both intervention
Cortez-Cooper et al. (2005) [48]	N = 23; aged = 29 ± 1.0 yo; BMI = NA	Resistance training	12 movements × 3–6 sets (5–10 reps) for 11 weeks	Light-day/heavy-day periodised approach (graded intensity)	Increase in PWV Rest: 791 ± 88 cm/s; post exercise: 833 ± 96 cm/s

IHG: Isometric handgrip exercise; RM: One-repetition maximum; HRmax: Maximal heart rate; HRR: Heart rate reserve; PWV: Pulse wave velocity; ASI: Arterial Stiffness Index.

**Table 4:** Characteristics of selected experimental trials in old women (n = 22).

Reference	Subjects’ Characteristics	Exercise Prescription			Main Findings
		Type	Time/Frequency	Intensity	
Pekas et al. (2020) [49]	Combined resistance and aerobic exercise group; N = 57; aged = 75 yo; BMI = 23.0 ± 4.0 mkg^−2^; Sedentary group; N = 44; aged = 78; BMI = 25.0 ± 3.0 mkg^−2^;	Combined resistance training and aerobic exercise	8 movements × 3 sets (10–15 reps) and 30 min walking/jogging/cycling × 3 days/week for 1 year.	Resistance training 12–15 RPE and 50–60% HRR for walking/jogging/cycling.	Decrease in PWV in exercise group. Exercise group: 12.1 ± 2.0 m/s; Sedentary group: 12.8 ± 1.8 m/s
Jaime et al. (2019) [50]	N = 33; aged = 65 ± 4.0 yo; BMI = 23.3 ± 2.6 mkg^−2^; Resistance training vs. Vibration training.	Resistance training vs. Vibration training	Resistance training; 4 movements × 1 set (15 rep) × daily for 12 weeks. Vibration training; 4 movements × 2–3 sets daily × 12 weeks.	Resistance training at 40% 1 RM, Vibration training at 24–40 Hz.	No change in PWV for both groups. Resistance: Rest: 11.7 ± 0.7 m/s; post exercise: 11.6 ± 0.7 m/s Vibration: Rest: 11.0 ± 0.4 m/s; post exercise: 10.6 ± 0.4 m/s
Molisz et al. (2019) [51]	Regular physical group; N = 38; aged = 59.4 yo; BMI = 24.1 mkg^−2^; and Control group; N = 17; aged = 62.4 yo; BMI = 24.9 mkg^−2^_._	Aerobic exercise for the Regular physical group and no exercise for the Control group.	Retrospective record of physical activity; 10 months record; at least 2 days/week of 1 hr session.	Low intensity	Decrease in PWV for Regular physical group. Exercise group: 7.4 m/s; Control group: 8.4 m/s
Kim et al. (2018) [52]	Aquatic group N = 14; aged = 66.77 ± 3.1 yo; BMI = NA, vs. Land-based group N = 14; aged = 67.42 ± 1.8 yo; BMI = NA.	Aquatic and land-based aerobic exercise.	Aquarobic 60 min × 2 days/week, 16 weeks vs., Aerobic (land-based) 60 min × 2 days/week for 16 weeks.	Graded intensity: (From 40–50% HRR to 65–70% HRR)	Decrease in PWV for Aquatic and Land-based groups
Nishiwaki et al. (2018) [53]	N = 21; aged = 76 ± 1.0 yo; BMI = 22.2 ± 0.7 mkg^−2^	Aerobic exercise, chair-based exercise.	60 min × 1 day/week for 8 weeks.	1.5–3.0 METs	Decrease in CAVI Rest: 9.2 ± 0.2 m/s; post exercise: 9.0 ± 0.2 m/s
Yasuda et al. (2016) [54]	Low-intensity elastic band BFR training group; N = 10; aged = 70 ± 6.0 yo; BMI = 20.8 ± 2.5 mkg^−2^; Middle-to high-intensity elastic band BFR training; N = 10; aged = 72 ± 7.0; BMI = 20.9 ± 2.1 mkg^−2^; Control group; N = 10; aged = 68 ± 6; BMI = 22.3 ± 2.8 mkg^−2^;	Resistance training with 50–200 mmHg blood flow restricted.	Low-intensity BFR group, 2 movements × 75 reps each. Middle–High intensity BFR group, 2 movements × 37–38 reps each × 2 days/week for 12 weeks.	Low-intensity BFR group used 5 OMNI resistance. Middle–High BFR group used 5.6 OMNI resistance.	No change in CAVI Rest: 8.4 ± 0.9 m/s; post exercise: 8.5 ± 0.8 m/s for both groups
Yasuda et al. (2015) [55]	N = 14; aged = 61–85 yo; BMI = NA	Resistance training (BFR)	2 movements × 75 reps × 2 days/week for 12 weeks	Submaximal bilateral arm curl and triceps press down exercise	No change in CAVI Rest: 9.1 ± 1.3 m/s; post exercise: 9.3 ± 1.1 m/s
Lee & Lee (2014) [56]	City-walking group; N = 19; aged = 71.11 ± 5.8 yo; BMI = 23.18 ± 2.7 mkg^−2^; Forest-walking group; N = 43, aged = 70.19 ± 4.66 yo; BMI = 24.32 ± 4.75 mkg^−2^;	Aerobic exercise (Forest-walking and city-walking)	60 min	Self-pace walking with normal breathing and without sweating, becoming over heated or experiencing palpitations	Forest-walking: Decrease in CAVI Rest: 8.32 ± 1.22 m/s; post exercise: 7.90 ± 1.09 m/s City-walking: no change in CAVI Rest: 8.59 ± 0.98 m/s; post exercise: 8.70 ± 0.86 m/s
Matsubara et al. (2014) [57]	Control group; N = 8; aged = 62 ± 3 yo; BMI = 23.0 ± 1.3 mkg^−2^; Aerobic exercise group; N = 11; aged = 62 ± 2 yo; BMI = 23.8 ± 0.6 mkg^−2^;	Aerobic exercise	40–60 min cycling and walking, >3 days/week for 12 weeks	70–80% HRmax	Increase in arterial compliance
Tanahashi et al. (2014) [58]	Exercise group; N = 20; aged = 62 ± 6 yo BMI = 22.5 ± 3.1 mkg^−2^; Control group; N = 10; aged = 61 ± 7 yo BMI = 23.1 ± 3.4 mkg^−2^;	Aerobic exercise	40–60 min/day, 3–6 days/week), for 12 weeks	65%–80% HRmax	Increase in arterial compliance
Rossow et al. (2014) [21]	N = 13; aged = 57 ± 3 yo; (postmenopausal) BMI = NA	Resistance training	6 movements × 8–10 reps × 2 sets and final set till failure × 3 days/week for 8 weeks	At 80% of 1 RM	No change in PWV Rest: 7.9 ± 1.4 m/s; post exercise: 7.5 ± 1.0 m/s
Corrick et al. (2013) [59]	N = 79; aged = over 60; BMI = NA divided into Group 1, N = 27; aged = 65.6 ± 0.7 yo; Group 2, N = 30; aged = 63.7 ± 0.5 yo, Group 3, N = 22; aged = 64.8 ± 0.7 yo.	Combined resistance training and aerobic exercise	Aerobic training for 40 min of cycling or running. Resistance training with 10 movements × 2 sets × 10 reps Groups 1, 1 day/week, Groups 2, 2 days/week, Group 3, 3 days/week, All carried out for 16 weeks	80% MHR and 80% 1 RM for aerobic and resistance training respectively.	Increase in arterial elasticity Group 1: Rest: 13.3 ± 0.9 m/s; post exercise: 15.3 ± 1.2 m/s Group 2: Rest: 13.8 ± 1.2 m/s; post exercise: 12.8 ± 0.7 m/s Group 3: Rest: 11.9 ± 0.7 m/s; post exercise: 13.1 ± 1.0 m/s
Lu et al. (2013) [60]	Interest class group; N = 16; aged = 68.9 ± 5. 8 yo; BMI = 24.8 ± 3.3 mkg^−2^; Tai Chi group; N = 15; aged = 73.9 ± 6.6 yo; BMI = 24.6 ± 3.1 mkg^−2^;	Aerobic exercise (Tai Chi training)	Interest class group; 60-min, 3 days/week, 16 weeks non-exercise activity.	NA	Increase in arterial compliance. Interest class group: Rest: 10.1 ± 2.7 mL/mmHg × 100; post exercise: 9.6 ± 3.8 mL/mmHg × 100 Tai Chi group: Rest: 10.3 ± 2.7 mL/mmHg × 100; post exercise: 13.0 ± 3.8 mL/mmHg × 100
Williams et al. (2013) [61]	Cross-over design, N = 22; aged = 66.7 ± 4.3 yo; BMI = 28.0 ± 4.6 mkg^−2^.	Resistance training and Flexibility training	4–5 movements × 2–3 sets × 8–12 reps × 2 days/week for 16 weeks. For stretching, 12 movements × 2 sessions/weeks for 16 weeks.	Resistance training: 8–12 RM	Female decrease in arterial stiffness
Miyaki et al. (2012) [62]	Total of, N = 22; Exercise group; Aged = 60 ± 6 yo; BMI = 22.2 ± 2.0 mkg^−2^; Control group; Aged = 60 ± 7 yo; BMI = 22.4 ± 2.6 mkg^−2^;	Aerobic exercise	30–45 min × 5 days/week for 2 months	60–75% HRmax	Decrease in β-stiffness index Exercise group: Rest: 8.72 ± 2.05; post exercise: 7.76 ± 1.97 Control group: Rest: 7.58 ± 1.34; post exercise: 7.71 ± 1.51
Ohta et al. (2012) [63]	Bench step exercise group; N = 13; aged = 72.2 ± 4.2 yo; BMI = 23.0 ± 2.6 mkg^−2^; Control group; N = 13; aged = 71.5 ± 7.4; BMI = 21.8 ± 2.6 mkg^−2^;	Aerobic exercise (Bench step exercise)	10–20 min × 40 steps/min with 10 steps increment every min × 3 times/day × 3 days/week for 12 weeks.	Lactate threshold.	Decrease in PWV Decreased by 206 ± 165.5 cm/s in exercise group.
Coelho et al. (2011) [64]	rs4646994 gene deletion/deletion group; N = 10; aged = 70.6 ± 5.8; BMI = 25.4 ± 3.2 mkg^−2^; rs4646994 gene insertion/insertion + insertion/deletion group; I/I + I/D group; N = 15; aged = 71.1 ± 6.5; BMI = 25.1 ± 2.4 mkg^−2^.	Aerobic exercise vs. no exercise.	20 min cycling session.	90% anaerobic threshold.	Lower AASI in the rs4646994 gene deletion/deletion group after cycling.
Figueroa et al. (2011) [65]	Exercise group; N = 12; aged = 54 ± 2 (postmenopausal) BMI = 24.2 ± 0.7 mkg^−2^. Control group; N = 12; aged = 54 ± 1; (postmenopausal) BMI = 23.1 ± 0.7 mkg^−2^.	Combined resistance training and aerobic exercise.	40 min total; for resistance 9 movements × 12 reps for 20 min; and for aerobic exercise; treadmill waling for 20-min. All exercises were carried out 3 days/week for 12 weeks.	60% 1 RM for Resistance training and 60% HRmax for aerobic exercise.	Decrease in PWV in exercise group. Decreased 0.8 ± 0.2 m/s in exercise group.
Nishiwaki et al. (2011) [66]	N = 16; aged = 56 ± 1 (postmenopausal) BMI = NA.	Aquatic aerobic with normoxic (749.3–750.0 mmHg) and hypoxic (600.1–603.8 mmHg) conditions.	30 min exercise × 4 days/week for 8 weeks.	50% VO_2_peak	Normoxic: No changes in PWV. Hypoxic: Decrease in PWV.
Miura et al. (2008) [67]	Control group; N = 23; aged = 68.9 ± 7.5; BMI = 23.7 ± 3.0 mkg^−2^; Exercise with 1 day/week group; N = 29; aged = 69.0 ± 6.5; BMI = 22.8 ± 2.4 mkg^−2^; Exercise with 2 day/week group; N = 25; aged= 69.5 ± 7.0; BMI = 23.5 ± 2.7 mkg^−2^;	Combined resistance training and aerobic exercise	90 min exercise × 1 day/week and another group 2 days/week for 12 weeks. For resistance, 6–8 movements × 15–20 reps × 3–5 sets for 12 weeks. For aerobic, 20 min cycling; and Chair-based exercise for 30-min.	Resistance training, lightweight dumbbells (500–1000 g), and Aerobic exercise at 70–75% HRmax.	1 day/week: group showed no change in PWV. Rest: 1597.6 ± 201.5 cm/s; post exercise: 1570.5 ± 208.1 cm/s 2 day/week group showed a decreased PWV. Rest: 1598.2 ± 165.6 cm/s; post exercise: 1473.1 ± 188.4 cm/s
Casey et al. (2007) [68]	Resistance training group; N = 13; aged = 58.7 ± 4.5 (postmenopausal) BMI = 25.5 ± 3.2 mkg^−2^; Aerobic exercise group; N = 10; aged = 59.7 ± 6.5 (postmenopausal) BMI = 27.1 ± 4.9 mkg^−2^;	Resistance training or Aerobic exercise.	40 min × 2 day/week for 18 weeks. For resistance training, 10 movements × 12 reps × 1 set. For aerobic, treadmill walking.	Resistance training at 50% 1 RM. Aerobic exercise at 65–80% HRR.	Resistance training showed no change in aortic augmentation index (AI_a)_, Rest: 28.9 ± 1.9; post exercise: 28.5 ± 1.9% Aerobic training showed decreased AI_a_. Rest: 28.8 ± 2.1; post exercise: 25.1 ± 1.4%
Sugawara et al. (2004) [69]	Total of N = 15 Low-intensity exercise training group; Aged = 58.0 ± 4.0 (postmenopausal) BMI = NA. Moderate-intensity exercise training group; Aged = 59.0 ± 6.0 (postmenopausal) BMI = NA	Aerobic cycling exercise	3–5 days/week 12 weeks.	Low-intensity group at 40% HRR, and Moderate-intensity group at 70% HRR.	Increase in arterial compliance in both groups. Low-intensity exercise training group: Rest: 0.7 ± 0.32 mm^2^/mmHg × 10^−1^; post exercise: 1.06 ± 0.55 mm^2^/mmHg × 10^−1^ Moderate-intensity exercise training group: Rest: 0.82 ± 0.37 mm^2^/mmHg × 10^−1^; post exercise: 1.14 ± 0.39 mm^2^/mmHg × 10^−1^

HRR: Heart rate reserve; RM: One-repetition maximum; HRmax: Maximal heart rate; PWV: Pulse wave velocity; BFR: Elastic band training with blood flow restriction; METs: Metabolic Equivalent; CAVI: Cardio-ankle vascular index; AASI: Ambulatory Arterial Stiffness Index; AI_a_: aortic augmentation index.

## Data Availability

Not applicable.
